# The line between COVID-19 pandemic and rare bone diseases

**DOI:** 10.1007/s11845-020-02400-6

**Published:** 2020-11-02

**Authors:** Luca Sangiorgi, Evelise Brizola, on behalf of the COVID-19 Helpline for Rare Bone Diseases Group, Andrea Vianello, Anna Zambrano, Annalisa Scopinaro, Davide Gatti, Elena Pianigiani, Elio Castagnola, Giampiero I. Baroncelli, Giovanna Mantovani, Giovanni Adami, Giuseppe Zampino, Laura Ruzzini, Laura Trespidi, Leonardo Panzeri, Lorena Casareto, Luigi A. Nasto, Marco Carbone, Maria Beatrice Michelis, Maria Francesca Bedeschi, Maria Luisa Brandi, Mauro Celli, Michaela V. Gonfiantini, Paolo Fraschini, Sandro Giannini, Silvio Boero, Stefania Sella, Vittorio Landoni

**Affiliations:** 1 Department of Rare Skeletal Disorders, IRCCS Istituto Ortopedico Rizzoli, Via Pupilli 1, 40136 Bologna, Italy; 2 CLIBI Laboratory, IRCCS Istituto Ortopedico Rizzoli, Bologna, Italy

The recent outbreak of COVID-19 pandemic had a dramatic global impact for healthcare systems and required a rapid rearrangement of the priorities. In Europe, a disease is defined as rare when it affects no more than 5 in 10,000 people [1]. Between 6000 to 8000 distinct rare diseases exist today affecting around 6–8% of the population—over 30 million people in Europe are directly involved [2], which number is close to the number of people currently affected with COVID-19 globally (more than 21 million) [3]. Among European countries, Italy has been particularly overwhelmed by COVID-19, with approximately 254,000 infected people and more than 35,000 deaths (WHO report of August 16th 2020) [3]. Suddenly, healthcare workers have found themselves in the front line of this historical moment of emergency [4], and at the same time, their patients started to feel helpless and abandoned, facing delays in medical visits or pre-scheduled surgeries and struggling in refilling even simple pharmacological prescriptions. Patients with rare bone diseases (RBD) have been in need of special care in this emergency situation, starting from seeking access to care and high-quality information. In fact, patients with RBD need careful attentions from a multidisciplinary team since complications can affect different organs and systems (i.e. the musculoskeletal, the pulmonary, the cardiovascular systems).

The priority for experts of RBD has become finding a way to communicate directly with patients and healthcare professionals (who often did not have any previous experience with patients with rare diseases). Thus, the European Reference Network on Rare Bone Diseases (ERN BOND) coordination team and some Italian RBD healthcare professionals have set up a direct and dedicated 24/7 telephone line the “COVID-19 Helpline for Rare Bone Diseases” [5]. The purpose is to provide experience and knowledge about RBD to patients and healthcare professionals working in the intensive care units and/or COVID-19-devoted wards who are treating or will treat patients affected by RBD, initially focusing on patients with osteogenesis imperfecta. For all patients with RBD, it is crucial to know that they can constantly rely on their primary care physicians and keep these professionals informed about their disease, in particular for those patients who walk with difficulty or cannot even walk independently and already have many other health complications related to their disease.

ERN BOND includes 38 centres from 10 European countries, and the network is actively sharing information to disseminate the best practices for COVID-19 cases and discuss the possibility to make the Helpline initiative available in other participating European countries as well. Considering that Spain, Germany, France and other European countries have also been hit by COVID-19, it is imperative to keep a highly collaborative profile among all countries in order to control the pandemic crisis. For this reason, many helpful initiatives have been implemented both in Italy and across Europe. Further information can be found on the ERN BOND website: http://ernbond.eu/

To date, more than 300 telephone calls and messages have been handled by the COVID-19 Helpline for RBD, and information about cardiopulmonary conditions, orthopedic problems, pharmacological interactions and treatments and other issues were carried out both to healthcare professionals and patients. In some cases, we have followed up patients by remote checking daily for negative evolutions and supporting general practitioners in the decision for referring (or not) patients to the COVID-19 wards.

We have also been frequently contacted by patients affected by rare diseases other than RBD; this clearly demonstrates that dedicated Helplines should be considered valuable and simple tools for reaching out who is in need of specific expertise in these extraordinary situations.

We would like to highlight that in this exact moment, there are more than 761,000 deaths caused by COVID-19 and more than 21,294,000 positive confirmed cases worldwide [3], and according to the general population data, approximately 6–8% of them might be affected by rare diseases. Our successful experience underlines the importance of organizing rapidly concrete initiatives aimed to provide information and expertise remotely to patients and healthcare professionals.

## Data Availability

Not applicable.
